# Understanding variation in catastrophic health expenditure from socio-ecological aspect: a systematic review

**DOI:** 10.1186/s12889-024-18579-7

**Published:** 2024-06-05

**Authors:** Kaniz Fatima Mohsin, Md. Nasif Ahsan, Mohammed Ziaul Haider

**Affiliations:** Economics Discipline, Khulna University, Khulna, Bangladesh

**Keywords:** Catastrophic Health Expenditure, Socio-ecological, Out-of-Pocket payment

## Abstract

**Background:**

Out-of-pocket (OOP) payment is one of many countries’ main financing options for health care. High OOP payments push them into financial catastrophe and the resultant impoverishment. The infrastructure, society, culture, economic condition, political structure, and every element of the physical and social environment influence the intensity of financial catastrophes in health expenditure. Hence, the incidence of Catastrophic Health Expenditure (CHE) must be studied more intensively, specifically from regional aspects. This systematic review aims to make a socio-ecological synthesis of the predictors of CHE.

**Method:**

We retrieved data from Scopus and Web of Science. This review followed PRISMA guidelines. The interest outcomes of the included literature were the incidence and the determinants of CHE. This review analyzed the predictors in light of the socio-ecological model.

**Results:**

Out of 1436 screened documents, fifty-one met the inclusion criteria. The selected studies were quantitative. The studies analyzed the socioeconomic determinants from the demand side, primarily focused on general health care, while few were disease-specific and focused on utilized care. The included studies analyzed the interpersonal, relational, and institutional predictors more intensively. In contrast, the community and policy-level predictors are scarce. Moreover, neither of the studies analyzed the supply-side predictors. Each CHE incidence has different reasons and different outcomes. We must go with those case-specific studies. Without the supply-side response, it is difficult to find any effective solution to combat CHE.

**Conclusion:**

Financial protection against CHE is one of the targets of sustainable development goal 3 and a tool to achieve universal health coverage. Each country has to formulate its policy and enact laws that consider its requirements to preserve health rights. That is why the community and policy-level predictors must be studied more intensively. Proper screening of the cause of CHE, especially from the perspective of the health care provider’s perspective is required to identify the individual, organizational, community, and policy-level barriers in healthcare delivery.

## Background

Health expenditure becomes catastrophic if the out-of-pocket (OOP) payment for healthcare exceeds a specified threshold, which can be determined based on income or ability to pay [1]. Measuring the Catastrophic Health Expenditure (CHE) may seem simple, but the outcome is challenging. High OOP makes health care inaccessible to needy people and may result in impoverishment. Impoverishment occurs when a health event compels a household to divert the expenditure on basic needs to such an extent that the spending on basic needs is reduced below the poverty line [2]. The incidence of CHE is showing a rising trend. Lots of factors are responsible for that rise. Disease prevalence is in a transitional phase, with non-communicable disease (NCD) spreading at an alarming rate due to changes in lifestyle [3]. Communicable diseases are still not controlled in many parts of the world, and climatic factors also change the disease type and severity [4]. The prevalence of NCD is increasing in middle and low-income countries, putting pressure on already stretched health systems [5]. If we consider the physical and financial burden of NCDs, they have a significant negative impact at the household level [6]. This is because OOP payment is the main financing option for most low- and middle-income countries to pay for health care [4]. According to a study, globally, 150 million people are facing CHE, with 90% coming from low-income countries [7]. According to the global monitoring report on tracking universal health coverage by the World Health Organization (WHO), each year, CHE causes 100 million people to become impoverished [8].

As per World Bank data, the global average of OOP expenditure is 16.36% of total health expenditure. For high-income groups, the average OOP expenditure is 12.12%, while for Lower Middle-Income Countries (LMIC), OOP expenditure is 41.96%. The gap is larger if we consider the country-specific data. Being part of the same sub-continent, OOP expenditure in Bangladesh is 74%, while in India, it is 50.59%, and in Pakistan, it is 55.44%. If we compare the situation with African nations, OOP expenditure in Ghana is 33.44%, while in Nigeria, it is 74.68% (World Bank, 2023). Like OOP, the incidence of CHE also varies across the region. According to WHO, the global average CHE incidence is 13%, suggesting that 13% of the world’s population has to cut off their consumption expenditure to pay for health. Whereas for LMICs, the incidence of CHE is 16%. Among the countries in the Indian subcontinent and sub-Saharan Africa, there is a significant difference in the incidence of CHE. In India, the incidence of CHE is 17%, whereas in Bangladesh, the percentage is 24. CHE incidence in Nigeria is 16%, whereas in Ghana and Rwanda, CHE incidence is only 1% (WHO, 2023). African nations are mostly war-torn and poverty-ridden countries. However, the countries in South Asia are relatively politically stable regions. Still, African nations are making progress in saving people from CHE. Despite their close geographical proximity and socioeconomic and cultural resemblance, significant variations exist across the countries. CHE needs to be understood more comprehensively to find the reason for this variation.

A systematic review combines evidence from existing literature with a focus on structure and methodology. A review of CHE is common in the available literature; nonetheless, the authors highlight the incidence and determinants. However, there is a dearth of synthesis of the predictors of CHE with the socio-ecological model in the existing literature. Synthesizing the predictors with the socio-ecological model will help to identify the gaps more precisely. Detection of the gaps will facilitate underlining the loopholes both from the demand and supply sides, based on which the policymakers would devise policy and bring necessary modifications to the existing health system. The WHO South-East Asia Journal of Public Health reported that financial protection is a global priority outlined in Sustainable Development Goal (SDG) 3 [9]. Most of the LMICs are lagging in providing financial protection against CHE. The reasons for CHE have to be analyzed in a more meaningful way.

### The guiding framework: the socio-ecological model

When confronted with a health issue, seeking medical assistance is contingent upon the individual’s social and environmental context. Whether a patient will encounter a qualified medical professional or an unqualified individual depends on various factors, including the patient’s knowledge, beliefs, attitudes, familial environment, and the characteristics of the healthcare system. Sarker et al. [10] concluded that this phenomenon can attribute to a lower OOP expenditure associated with a higher prevalence of CHE, which is frequently observed in most LMICs, where it is challenging to ensure the physical accessibility of healthcare services to patients requiring them.

Nevertheless, without addressing these socioeconomic, religious, and cultural concerns, making health care physically available alone would not ensure accessibility. The physical environment, including ecological and natural phenomena and the social environment in which individuals undergo their developmental years, influence health [11]. Urie Bronfenbrenner introduced the socio-ecological model in 1979 to explain how the surrounding ecology and socioeconomic environment influence a child’s development [12]. Further, McLeroy and colleagues updated the model to explain health-seeking behavior and identified five levels influencing health behavior, practices, and conditions [13]. These five levels are interpersonal, relational, institutional, community, and policy [14]. Interpersonal factors encompass personal attributes, knowledge, and beliefs governing people’s behavior and practices. The relational factors encompass the impact of familial dynamics, peer relations, and social networks. Institutional factors like educational institutions, diverse commercial enterprises, religious institutions, and healthcare management, substantially influence individuals’ proclivity to pursue healthcare services. Community-level factors primarily encompass the dynamic interplay between institutional factors and organizations. Policy-level factors contain laws and regulations at the international, national, and local levels that govern healthcare administration and other affiliated entities. The model comprehensively incorporates various socioeconomic, political, and cultural dimensions.

**Fig. 1:**
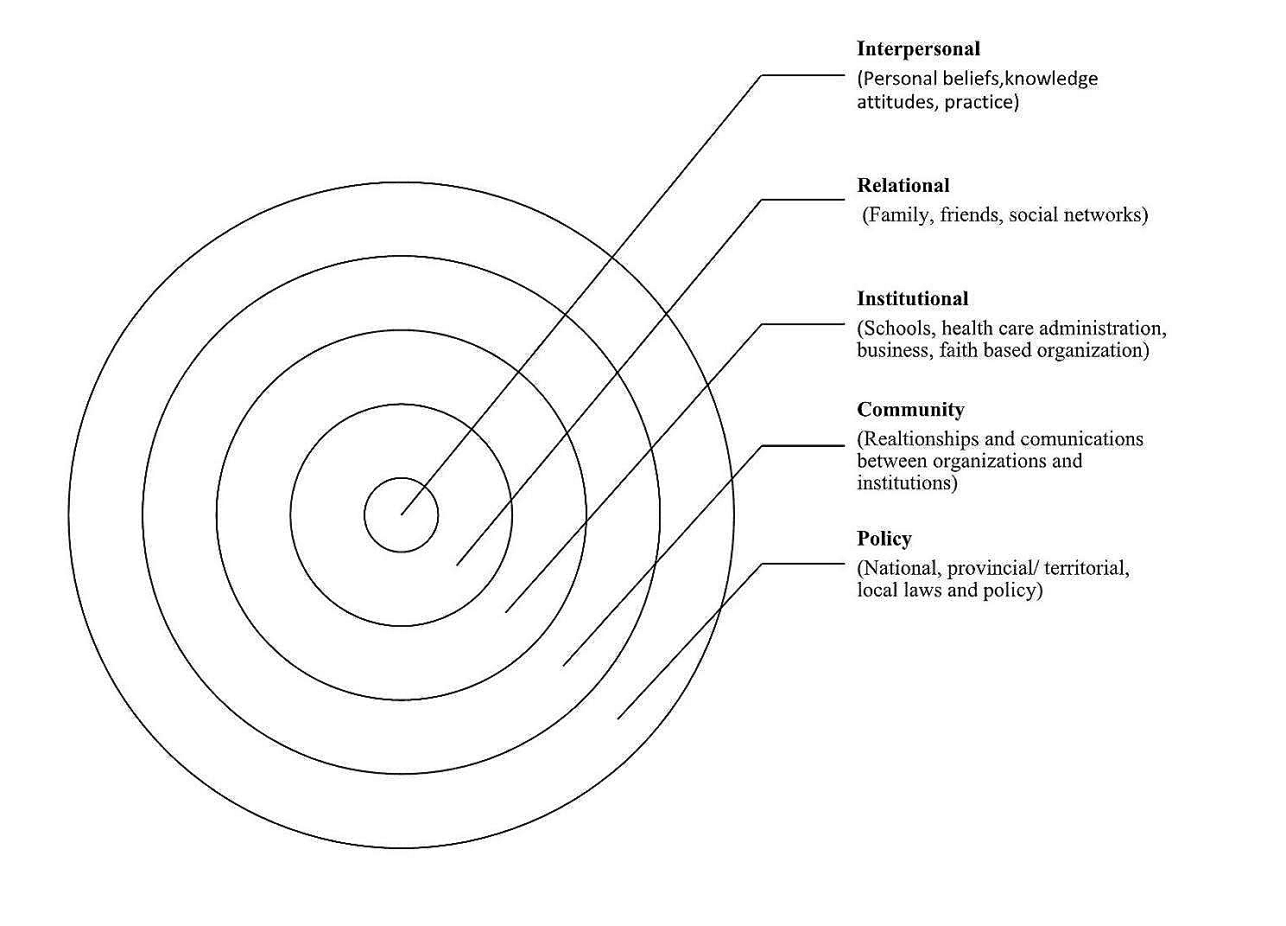
Socio-ecological model, Adapted from McLaren L, Hawe P. Ecological perspectives in health research. Journal of epidemiology and community health. 2005 Jan; 59 [15]:6

This paper intends to synthesize the predictors of CHE with the socio-ecological model to find out the gap in the existing literature. Figure 1 presents the main themes. This paper first sorted out the predictors of CHE, then categorized the predictors according to the five levels of the socio-ecological model: interpersonal, relational, intuitional, community, and at the policy level, and finally tried to find out which level of the model has been less focused, where is the gap. Pulling the poor out of CHE requires a holistic effort, and all the loopholes in the health system must be identified carefully from each level.

## Methods

**Fig. 2:**
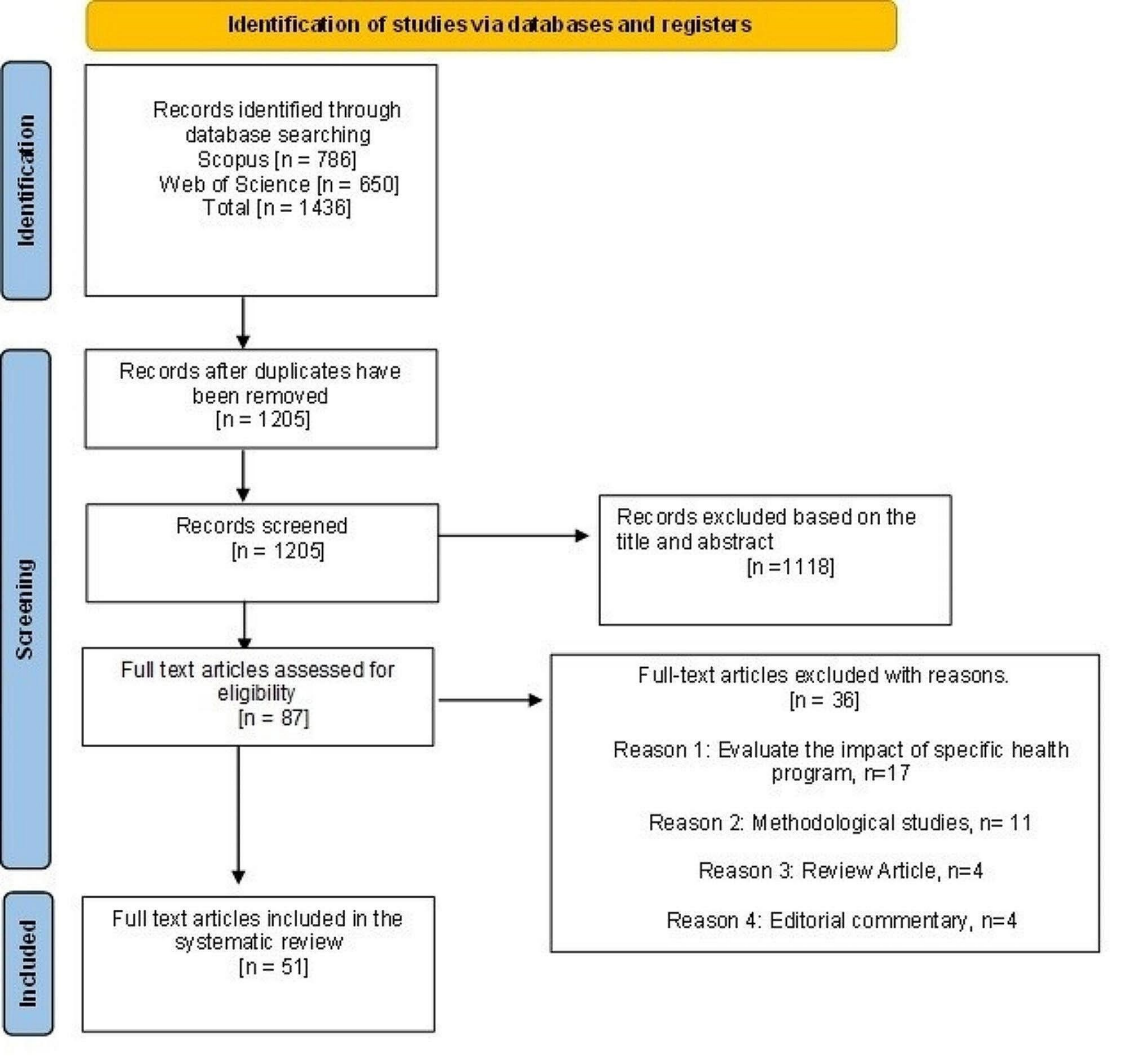
PRISMA flow chart

This review followed the Preferred Reporting Items for Systematic Reviews and Meta-Analysis (PRISMA) guideline in deciding the mandatory components of a systematic review. Figure 2 presents the sequential flow chart of screening the literature following PRISMA.

### Search strategy and inclusion criteria

We retrieved data from Scopus and Web of Science electronic databases. The time frame of the included publications was from 2011 to 2023. We did a systematic search with the combination of text words with the thesaurus terms ‘catastrophic health expenditure’ with ‘incidence’, ‘impact’, ‘impoverishment’, ‘determinant’, ‘predictors’, ‘economic impact’, ‘financial burden’, ‘coping mechanism, and ‘source of finance’. The subject area of the search was confined to health, biomedical science, arts, and social science, and the language was restricted to English. We present the inclusion and exclusion criteria in Table 1.

**Table 1:** Inclusion and exclusion criteria

Factor	Inclusion Criteria	Exclusion Criteria
**Purpose**	Focus on the incidence of catastrophic health expenditure and their determinants	Study on program evaluation or estimation of methodological appropriateness
**Publication type**	Published full text article	Unpublished source, review article, editorial commentary
**Review status**	Peer reviewed	Non-peer-reviewed sources
**Language**	English	All languages apart from English
**Focusing country**	Low- and middle-income countries as per the world bank criteria	Study on high-income country as per the world bank criteria
**Subject area**	Health, biomedical science, arts, and social science	Business and Management

### Data extraction and analysis

Consulting with the co-authors, the second author extracted the data. With the search key, 1,436 documents were identified. The inclusion and exclusion criteria were decided based on the discussion of the three authors. Analyzing the title and abstract, the first and second authors excluded 1,118 documents as per the exclusion criteria. We finally selected eighty-seven documents for full-text review in consultation with the third author. The first and second authors reviewed eighty-seven documents for full-text assessment. After assessing eighty-seven documents, thirty-six articles were excluded, seventeen focused on some health program evaluation, twelve documents evaluated the methodological appropriateness, four were review articles, and four documents were editorial commentary. Assessing the full text, three authors finally agreed to include fifty-one articles in the review.

The primary focused information includes the study context (i.e., publication year and country), features of the study population, and methodology (i.e., data source, research approach, study focus, utilized care). The focus of the included literature was the determinants of CHE.

Fifty-one studies from fifteen countries have been analyzed, where Bangladesh (sixteen documents) and China (eleven documents) had dominations. Among the sixteen papers from Bangladesh, seven studies used Household Income and Expenditure Survey data. China used different databases as they have versatile data sources like the China Health and Retirement Longitudinal Study, National Health Service Survey, China Family Panel Studies, and Health Services Survey in different provinces. Five studies used the China Health and Retirement Longitudinal Study, which depicts China concentrating on the age-specific health needs of its population, as age is a strong predictor of health status and CHE. Bangladesh has no age-specific database, so their studies do not consider age-differential health needs.

## Results

### Quality appraisal of the included literature

The quality of the included articles has been assessed following the Crombie critical appraisal guideline. The first two authors independently assessed the quality of the included literature. This is a tool used to appraise the quality of quantitative analysis using cross-sectional data. All the included literature was quantitative and mainly used cross-sectional data. The procedure contains 11 questions presented in Table 2 with three probable responses: ‘Yes,’ ‘No,’ and ‘Can’t tell.’ Among the 11 criteria, the included pieces of literature fulfilled 6–10 criteria. A single study met ten criteria; forty-three studies met nine criteria; six studies met seven criteria; and a single study met six criteria. Forty-seven studies used random sampling, whereas a single study used convenience sampling. Three documents did not report the sampling procedure. Sixteen documents reported the response rate. Based on the Crombie score, forty-four studies were of high quality, and the quality of seven studies was poor. Details of the critical appraisal have been presented in Table 2.

**Table 2:** Quality appraisal of the included literatures

SI	Author, Year, Country	Did the study address a clearly focused question/issue?	Is the study design appropriate for answering the research question?	Is the method of selection of the subjects (employee, teams, division, organizations) clearly described?	Could the way the sample was obtained introduce (selection) bias?	Was the sample of subjects representative with regard to the population to which the findings will be referred?	Was the sample size based on pre-study considerations of statistical power?	Was a satisfactory response rate achieved?	Are the measurements (questionnaires) likely to be valid and reliable?	Was the statistical significance assessed?	Are confidence intervals given for the main results?	Could there be confounding factors that haven’t been accounted for?	Can the results be applied to your organization?	Total Score and Quality
1.	Ahmed et al., 2021, Bangladesh	Y	Y	Y	N	N	Y	Y	N	Y	Y	N	Y	7/11 (63.6) Poor
2.	Ahmed et al., 2022, Bangladesh	Y	Y	Y	N	Y	Y	Y	Y	Y	Y	N	Y	9/11 (81.8) High
3.	Aregbeshola and Khan, 2018, Nigeria	Y	Y	Y	N	Y	Y	Y	Y	N	N	N	Y	7/11 (63.6) Poor
4.	Aregbeshola and Khan, 2018, Nigeria	Y	Y	Y	N	Y	Y	Y	Y	Y	Y	N	Y	9/11 (81.8) High
5.	Azzani et al., 2017, Malaysia	Y	Y	Y	N	Y	Y	Y	Y	Y	Y	N	Y	9/11 (81.8) High
6.	Bashir et al., 2021, Pakistan	Y	Y	Y	N	Y	Y	Y	Y	Y	Y	N	Y	9/11 (81.8) High
7.	Begum and Hamid, 2021, Bangladesh	Y	Y	Y	N	Y	Y	Y	Y	N	N	N	Y	(7/11) (63.6) Poor
8.	Boing et al., 2014, Brazil	Y	Y	Y	N	Y	Y	Y	Y	N	Y	Y	Y	9/11 (81.8) High
9.	Buigut et al., 2015, Kenya	Y	Y	Y	N	Y	Y	Y	Y	N	Y	Y	Y	9/11 (81.8) High
10.	Choi et al., 2015, South Korea	Y	Y	Y	N	Y	Y	Y	Y	Y	Y	N	Y	9/11 (81.8) High
11.	Chowdhury et al., 2021	Y	Y	Y	N	Y	Y	Y	Y	Y	Y	Y	Y	10/11 (90.9) High
12.	Dalui et al., 2020, India	Y	Y	Y	N	Y	Y	Y	Y	Y	Y	N	Y	9/11 (81.8) High
13.	Dang et al., 2023, China	Y	Y	Y	N	Y	Y	Y	Y	Y	Y	N	Y	9/11 (81.8) High
14.	Datta et al., 2019, Pakistan	Y	Y	Y	N	Y	Y	Y	Y	Y	Y	N	Y	9/11 (81.8) High
15.	Dorjdagva et al., 2016, Mongolia	Y	Y	Y	N	Y	Y	Y	Y	Y	Y	N	Y	9/11 (81.8) High
16.	Duan et al., 2019, China	Y	Y	Y	N	Y	Y	Y	Y	Y	Y	N	Y	9/11 (81.8) High
17.	Yazdi-Feyzabadi et a.l, 2018, Iran	Y	Y	Y	N	Y	Y	Y	Y	Y	Y	N	Y	9/11 (81.8) High
18.	Fu et al., 2022, China	Y	Y	Y	N	Y	Y	Y	Y	Y	Y	N	Y	9/11 (81.8) High
19.	Fu e al., 2021, China	Y	Y	Y	N	Y	Y	Y	Y	Y	Y	N	Y	9/11 (81.8) 12.High
20.	Ghimire et al.,. 2018, Nepal	Y	Y	Y	N	Y	Y	Y	Y	Y	Y	N	Y	9/11 (81.8) High
21.	Haque et al., 2021, Bangladesh	Y	Y	Y	N	Y	Y	Y	Y	Y	Y	N	Y	9/11 (81.8) High
22.	Hoque et al., 2015, Bangladesh	Y	Y	Y	C	N	Y	C	N	Y	Y	N	Y	6/11(54.5) Poor
23.	Islam et al., 2021, Bangladesh	Y	Y	Y	Y	Y	Y	Y	N	N	N	N	Y	7/11 (63.6) Poor
24.	Jung 2021., South Korea	Y	Y	Y	N	Y	Y	Y	Y	Y	Y	N	Y	9/11 (81.8) High
25.	Khan et al., 2017, Bangladesh	Y	Y	Y	N	Y	Y	Y	Y	Y	Y	N	Y	9/11 (81.8) High
26.	Kien et al., 2016, Vietnam	Y	Y	Y	N	Y	Y	Y	Y	Y	Y	N	Y	9/11 (81.8) High
27.	Kim et al., 2011, South Korea	Y	Y	Y	N	Y	Y	Y	Y	Y	Y	N	Y	9/11 (81.8) High
28.	Koris et al., 2017, Malaysia	Y	Y	Y	N	Y	Y	Y	Y	Y	Y	N	Y	9/11 (81.8) High
29.	Liu et al., 2021, China	Y	Y	Y	N	Y	Y	Y	Y	Y	Y	N	Y	9/11 (81.8) High
30.	Mchenga., 2017, Malawi	Y	Y	Y	N	Y	Y	Y	Y	N	N	N	Y	7/11 (63.3), Poor
31.	Miao 2022., China	Y	Y	Y	N	Y	Y	Y	Y	Y	Y	N	Y	9/11 (81.8) High
32.	Misra et al., 2013, India	Y	Y	Y	N	Y	Y	Y	Y	Y	Y	N	Y	9/11 (81.8) High
33.	Mohanty et al., 2022, India	Y	Y	Y	N	Y	Y	Y	Y	Y	Y	N	Y	9/11 (81.8) High
34.	Molla et al., 2017, Bangladesh	Y	Y	Y	N	Y	Y	Y	Y	Y	Y	N	Y	9/11 (81.8) High
35.	Mutyambizi et al., 2019, South Africa	Y	Y	Y	N	Y	Y	Y	Y	Y	Y	N	Y	9/11 (81.8) High
36.	Mwai and Muriithi, 2016, Kenya	Y	Y	Y	N	Y	Y	Y	Y	Y	Y	N	Y	9/11 (81.8) High
37.	Rahman et al., 2022, Bangladesh	Y	Y	Y	N	Y	Y	Y	Y	Y	Y	N	Y	9/11 (81.8) (High)
38.	Rahman et al., 2013, Bangladesh	Y	Y	Y	N	Y	Y	Y	Y	Y	Y	N	Y	9/11 (81.8) (High)
39.	Rahman et al., 2022, Bangladesh	Y	Y	Y	N	Y	Y	Y	Y	N	N	N	Y	7/11 (63.6) (Poor)
40.	Sarker et al., 2021, Bangladesh	Y	Y	Y	N	Y	Y	Y	Y	Y	Y	N	Y	9/11 (81.8) High
41.	Sarker et al., 2022, Bangladesh	Y	Y	Y	N	Y	Y	Y	Y	Y	Y	N	Y	9/11 (81.8) High
42.	Sarker et al., 2018, Bangladesh	Y	Y	Y	N	Y	Y	Y	Y	Y	Y	N	Y	9/11 (81.8) High
43.	Sayuti and Sekuri, 2022, Malayasia	Y	Y	Y	N	Y	Y	Y	Y	Y	Y	N	Y	9/11 (81.8) High
44.	Sheikh et al., 2022, Bangladesh	Y	Y	Y	N	Y	Y	Y	Y	Y	Y	N	Y	9/11 (81.8) High
45.	Si et al., 2019, China	Y	Y	Y	N	Y	Y	Y	Y	Y	Y	N	Y	9/11 (81.8) High
46.	Thuong et al., 2020, Vietnam	Y	Y	Y	N	Y	Y	Y	Y	Y	Y	N	Y	9/11 (81.8) High
47.	Wang et al., 2023, China	Y	Y	Y	N	Y	Y	Y	Y	Y	Y	N	Y	9/11 (81.8) Hig
48.	Wang et al., 2015, China	Y	Y	Y	N	Y	Y	Y	Y	Y	Y	N	Y	9/11 (81.8) High
49.	Yadav et al., 2021, India	Y	Y	Y	N	Y	Y	Y	Y	Y	Y	N	Y	9/11 (81.8) High
50.	Zhao et al., 2022, China	Y	Y	Y	N	Y	Y	Y	Y	Y	Y	N	Y	9/11 (81.8) High
51.	Zhen et al., 2018, China	Y	Y	Y	N	Y	Y	Y	Y	Y	Y	N	Y	9/11 (81.8) High

### Characteristics of the included literature

Among the fifty-one documents, sixteen studies were from Bangladesh, eleven were from China, four were from India, and three were from South Korea and Malaysia each; two were from Kenya, Nigeria, Vietnam, and Pakistan. Single papers were selected from Brazil, Iran, Malawi, Mongolia, Nepal, and South Africa. The merit of selecting the literature from countries with different development statuses was to capture the variation in the predictors of CHE. Seventeen papers were primary data-based, and thirty-four papers were based on secondary data. The secondary data-based studies used sources like the National Database, Household Income and Expenditure Survey, Family Panel data, and Health and Retirement Longitudinal Study. Of eleven studies from China, nine papers used secondary data; out of sixteen papers from Bangladesh, nine used primary data. The study sample covered in the selected literature includes general households, the elderly (aged 65+), and persons with disabilities, children, and mothers. Twenty-three studies were disease-specific (encompassed NCD, cancer, diabetes, hypertension, chronic illness, chronic liver disease, spinal cord injury, rotavirus infection, diarrhea, and tuberculosis), whereas twenty-six studies concentrated on general health care, and two papers focused on multiple diseases. Forty-one studies used cross-sectional data, while seven studies used longitudinal data. Forty-six papers have considered both inpatient and outpatient care; three papers concentrated on the utilization of inpatient care, and two papers focused on medication and community-based home care. Detailed information, along with the predictors of CHE, has been summed up in Table 3.

**Table 3:** Characteristics of the included literature

SI	Author, Year, Country	Aims	Study Design	Sampling Method	Participants	Focused Health Problem	Health care utilization	Predictors of CHE
1.	Ahmed et al., 2021, Bangladesh	To assess the economic burden of Rotavirus Hospitalization of under-five children	Primary data, Cross-sectional,	Not Reported	546 (Under 5 children)	Rotavirus	Inpatient	Hospitalization cost, indirect cost like the income loss of the caregiver, food and lodging cost
2.	Ahmed et al., 2022, Bangladesh	To investigate the incidence of CHE and impoverishment from OOP payments and their determinants	Secondary data, (HIES 2016–2017), Cross-sectional,	Multistage, stratified random sampling	46,076 Households Response rate: 99.99%	General Health Care	Both inpatient and outpatient	Medicine cost, number of people aged 65+, type of family, rural-urban residence
3.	Aregbeshola and Khan, 2018, Nigeria	To measure the financial burden of OOP	Secondary data, Harmonized Nigeria Living Standard Survey (HNLSS), 2009/2010, Cross-sectional	Multistage random sampling	38,700 households	General health care	Both inpatient and outpatient	Economic status, type of provider
4.	Aregbeshola and Khan, 2018, Nigeria	To assess the determinants of catastrophic health expenditure	Secondary data The Harmonized Nigeria Living Standard Survey (HNLSS) 2009/10, Cross-Sectional	Stratified random sampling	38,700 Households	General health care	Both inpatient and outpatient	Economic status, gender, and number of people aged 65+, are weak predictors whereas the type of health care facility, type of illness, geopolitical zone, rural-urban residence, education, and sex of household head
5.	Azzani et al., 2017, Malaysia	To explore the prevalence and determinants of CHE	Primary data, Longitudinal	Universal sampling approach	138 patients Response rate: 98.5%	Colorectal cancer	Both inpatient and outpatient including surgery, chemotherapy, radiotherapy	Poverty, surgery,
6.	Bashir et al., 2021, Pakistan	To estimate the incidence and determinant of CHE	Secondary data Household Integrated Economic Survey, 2015–2016 and 2018–2019, Cross-sectional	Stratified random sampling	24,238 (2015–2016), 24, 809 (2018–2019)	General health care	Not reported	Income, gender, and education of household head, number of older adults (aged 65+) and children
7.	Begum and Hamid, 2021, Bangladesh	To explore whether the poverty impact of OOP varies across the region based on ecological diversity	Primary data, Cross-sectional	Multistage stratified sampling	4,200 Households ( Low- Disaster-Prone and High-Disaster-Prone Areas), Response Rate: 90.26%	General Health Care	Both inpatient and outpatient	The relative prevalence of disaster, type of disease, medicine cost, nonfunctioning public facilities, and absence of private facilities
8.	Boing et al., 2014, Brazil	To measure the incidence and inequality of CHE	Secondary data, National Household Budget Survey, 2002–2003, 2008–2009, Cross-sectional	Two-stage cluster sampling	1,04,440 households	General health care	Both inpatient and outpatient	Poverty, education
9.	Buigut et al., 2015,Kenya	Incidence and determinants of CHE among slums	Secondary data, Indicator Development for Surveillance of Urban Emergencies, Cross-section data	Cluster sampling	Not reported	General health care	Both inpatient and outpatient	HI(+), Hospitalized members, Members with chronic disease, inpatient care, high cost of private facilities, age, gender and education of household head, number of children, adult earning member, membership of any social safety net
10.	Choi et al., 2015, South Korea	Measure the burden of chronic disease and its association with CHE	Secondary data, The South Korea Health Panel Survey (KHPS),2008, Cross-sectional	two-stage stratified random cluster sampling	7,006 households, Response rate: 94.1%	Chronic disease	Both inpatient and outpatient	Income, number of older adults with chronic disease
11.	Chowdhury et al., 2021	To estimate the cost-of-illness of TB and the incidence of CHE, and their determinants.	Primary data ,Cross-sectional	Systematic random sampling	900 TB patients Response rate: 96.2%	Tuberculosis	Both inpatient and outpatient	Poverty, indirect cost of income loss of the patient and caregiver, delayed diagnosis, diagnostic and medicine costs, household size, and social stigma
12.	Dalui et al., 2020, India	To determine the magnitude of OOP and determinants of CHE	Primary Data, Cross-sectional	Multistage random Sampling	235 households, above 18 years old, Response rate: 90%	General health care	Both inpatient and outpatient	Type of provider, education, and gender of household head, health isurance
13.	Dang et al., 2023, China	Explore the prevalence, equity, and determinants of CHE	Secondary data, 6th Health Services Survey in Gansu Province, China, Cross-sectional	Multistage Random Sampling	270 households	Diabetes		Income, comorbidity, household size (+)
14.	Datta et al., 2019, Pakistan	To assess the relationship between OOP and CHE	Secondary data, Household Integrated Economic Survey, 2015–2016, Cross-sectional.	Stratified Two-stage Random Sampling	24,238 households	Blood pressure and diabetes	Medication ,	Lack of regulation, Sub performance of drug regulatory authorities
15.	Dorjdagva et al., 2016, Mongolia	To analyze the incidence of CHE and the impoverishment impact	Secondary data Household Socioeconomic Survey (HSES), 2012, Cross-sectional	Stratified random sampling	12,811 households	General health care	Both inpatient and outpatient	High OOP
16.	Duan et al., 2019, China	To investigate the extent and association of patents/diagnostic delay and other potential factors with CHE for tuberculosis (TB)	Primary data, Cross-sectional,	Stratified random sampling	1199 (Registered patients with Active pulmonary TB), Response rate: 92.9%	Tuberculosis	Both inpatient and outpatient	Patient’s / diagnostic delay, Cost of medicine and diagnosis, ancillary services for liver protection, health insurance, delayed or ignored inpatient treatment coverage from insurance,
17.	Yazdi-Feyzabadi et a.l, 2018, Iran	To measure the prevalence and intensity of CHE in Iran and to find out the determinant of CHE	Secondary data Eight national repeated cross-sectional survey run by Iran Statics Centre (ISC), Cross-sectional	Three stage stratified random cluster sampling	Sample size ranged from 36, 772 to 39,008 Households	General health care	Both inpatient and outpatient	Rural-urban residence, income, hospitalization, number of elderly older adults (65+)
18.	Fu et al., 2022, China	To investigate the prevalence of multi-morbidity and CHE	Secondary data National Household Survey, 2013, Cross-sectional	Multistage stratified cluster random sampling	8,471 individuals (Aged 18 years and above)	Diabetes	Both inpatient and outpatient	Income, Age, sex, education, employment status ( jobless, retired), chronic disease, rural-urban residence, health insurance
19.	Fu e al., 2021, China	To examine the association between multi-morbidity and CHE	Secondary data China Family Panel Studies (CFPS), 2012–2018, Panel Data	Three-stage stratified Probability-Proportional-to-Scale (PPS), Random Sampling	Not reported	NCD	Both inpatient and outpatient	Rural-urban residence, household income, education,
20.	Ghimire et al.,. 2018, Nepal	To investigate the cumulative incidence, distribution, and determinant of CHE in Nepal	Secondary data, Nepal living standard survey- third (2010/11), Cross-sectional	Stratified Random Sampling	5719 Households Response rate: 95.5%	General health care	Both inpatient and outpatient	Household member with chronic illness, higher episode of acute illness, income, rural-urban residence and education of household head
21.	Haque et al., 2021, Bangladesh	To assess the impact of OOP on liver disease, CHE impact, distress financing and their determinants	Primary data, Cross-sectional	Convenience sampling	107 hospitalized patients (Two public hospitals)	Chronic liver disease	Both inpatient and outpatient	Cost of medicine and diagnosis
22.	Hoque et al., 2015, Bangladesh	To measure the economic costs of maternal complication and their coping mechanism	Primary data, Cross-sectional	Not reported	706 woman with maternal complications (From 6 weeks to 6 months postpartum)	Maternal Complications	Both inpatient and outpatient	Method of delivery, rural-urban location, poverty, pregnancy-related complicacy
23.	Islam et al., 2021, Bangladesh	To find out the cost of providing community-based services, health care cost and economic burden of spinal cord injury	Primary data, Randomized Control Trial	Not reported	410 people with spinal cord injury	Spinal cord injury	Community-based care	Transport, food, accommodation cost, type of provider
24.	Jung 2021., South Korea	To determine the effect mid-to-long-term hospitalization (MLTH) on the incidence of CHE	Secondary data South Korean Welfare Panel Study, 2015,2016,2017, Cross-sectional	Systematic random sampling	1,671 Households, One member who was hospitalized for more than seven days	General health care	Inpatient	Earned income reduction (EIRR) (+)
25.	Khan et al., 2017, Bangladesh	investigated the impact of OOP payments. on CHE and poverty	Quantitative, Secondary Data (HIES 2010), Cross-sectional	Two-stage stratified random sampling	12,240 Households	General health care	Both inpatient and outpatient	Poverty, rural-urban residence
26.	Kien et al., 2016, Vietnam	To assess the socioeconomic inequalities in CHE and impoverishment	Primary data, Cross-sectional	Multistage cluster sampling	1020 household Response rate: 84.22%	NCD	Both inpatient and outpatient	NCD, number of elderly adults (Aged 65+)
27.	Kim et al., 2011, South Korea	To explore the relationship between household income and CHE	Secondary, The 2006 South Korean Household Income & Expenditure Survey, Cross-sectional	Two-stage stratified random cluster sampling	90, 696 households	General health care	Both inpatient and outpatient	Income, health insurance
28.	Koris et al., 2017, Malaysia	To investigate the socio-demographics, 15cognitive status an16d com17orbidities and ho18spital utilizatio19n factors that20 affect CHE21	Quantitative, Primary data, Cross-sectional	Multi-stage random Sampling	2274 (Aged 65+) Response rate: 97.9%	General health care	Both inpatient and outpatient	Age, rural-urban residence, ethnicity, income, and cancer prevalence
29.	Liu et al., 2021, China	To analyze the trends, incidence, intensity and determinants of CHE in China	Quantitative, Secondary data (China Family Panel Studies (CFPS)), Longitudinal	Multistage probability sampling	7386 Households	General health care	Both inpatient and outpatient	Age, education, self-rated health, chronic disease, number of elderly older adults(aged 65+), household size, income, hospitalization, rural-urban residence sex of household head, and health insurance.
30.	Mchenga., 2017, Malawi	To measure the incidence of CHE and the depth of poverty	Secondary data, The 3rd Malawi Integrated Household Survey (HIS-3), Cross-sectional	Two-stage stratified random sampling	12,271 Households	General health care	Both inpatient and outpatient	Income, rural-urban residence
31.	Miao 2022., China	To measure the determinants of CHE among middle-aged and older adults	Secondary data, China Health and Retirement Longitudinal Study (CHARLS), 2018	Multistage Stratified Probability sampling	9,186 households	Chronic disease	Both inpatient and outpatient	Poverty, malignant tumor, hospitalization, type of health insurance
32.	Misra et al., 2013, India	To measure the magnitude, distribution, and determinants of CHE in North India	Primary data, Cohort Study	Two-step cluster sampling	400 Households	General health care	Both inpatient and outpatient	Income, sickness days, and hospitalization
33.	Mohanty et al., 2022, India	To measure the determinants of CHE among middle-aged and older adults	Secondary data, Aging Study in India (LASI), 2017–2018, Longitudinal	Multistage Random Sampling	42,949 Households with at least one-member aged 45+	General health care	Both inpatient and outpatient	Income, poverty, old age dependency, rural-urban residence, ethnicity
34.	Molla et al., 2017, Bangladesh	To find out the determinants of high OOP on healthcare	Quantitative, Secondary data (HIES 2010), Cross-sectional	Multi-stage cluster sampling	10,701 Household, Response rate: 87.43%	General health care	Both inpatient and outpatient	Chronic disease, gender, health insurance
35.	Mutyambizi et al., 2019, South Africa	To investigate the incidence, socioeconomic inequality and determinants of CHE in South-Africa	Primary data, Cross-sectional	Stratified random sampling	405 (Diabetic patients aged above 21 taking care in two selected public tertiary level Diabetes Clinics), Response rate: 81%	Diabetes	Both inpatient and outpatient	Wealth, number of children, household size, social network, OOP, gender
36.	Mwai and Muriithi, 2016, Kenya	To measure the impoverishment impact of CHE	Secondary data, The 2007 Kenya Household Expenditure and Utilization Survey, Cross-sectional	Two-stage cluster sampling	39,795 individuals Response rate: 96%	NCD	Both inpatient and outpatient	NCD
37.	Rahman et al., 2022, Bangladesh	To investigate the trend and pattern of financial risk protection against NCD	Secondary data, (HIES 2005, 2010, 2016), Cross-sectional	2005,2010- Two-stage stratified random sampling, 2016- Two-stage stratified cluster sampling	2005- 10, 080 Households 2010- 12,240 Households 2016-46,076 Households	NCD	Both inpatient and outpatient	NCD, age, gender, medicine cost, unavailability of essential drugs at public facilities
38.	Rahman et al., 2013, Bangladesh	To investigates the determinants of high healthcare expenditure and healthcare- related financial catastrophe.	Primary data, Cross-sectional	Systematic random sampling	1,593 Household Response rate: 99.6%	Chronic illness	Both inpatient and outpatient	Hospitalization, chronic illness, poverty and education
39.	Rahman et al., 2022, Bangladesh	To explore the determinants of forgone care and financial burden of OOP n health	Secondary data (HIES 2016–2017), Cross-sectional	Stratified two-stage cluster sampling	39,124 Households, Response rate: 84.91%	General health care	Both inpatient and outpatient	Type of provider, chronic illness, household size, rural-urban residence
40.	Sarker et al., 2021, Bangladesh	To analyze the financial incidence of OOP on healthcare	Secondary data (HIES 2016–2017), Cross-sectional	Two-stage stratified random sampling	34,752 Individuals	General health care	Both inpatient and outpatient	Regressive OOP, poverty, rural-urban residence, cost of medicine, physician’s fee, unregulated market,
41.	Sarker et al., 2022, Bangladesh	To identify the determinants of OOP in urban citizens	Secondary data, Primary data, Cross-sectional	Two-stage cluster sampling	3,100 Household	Disease-specific (Multiple)	Both inpatient and outpatient	Poverty, rural-urban residence, regressive OOP
42.	Sarker et al., 2018, Bangladesh	To estimate and sex-specific economic cost of diarrheal disease	Primary data, Cross-sectional	Simple random sampling	801 pa	Diarrhea .	Both inpatient and outpatient	Age, indirect cost of patient and caregivers, poverty patient
43.	Sayuti and Sekuri, 2022, Malayasia	To assess the progress towards sustainable development goal 3.8.2 and determinants of CHE in Malaysia	Secondary data, (Household Expenditure Survey 2015/2016), Cross-sectional	Probability Sampling	13,015 Household, Response rate: 89.5%	General health care	Both inpatient and outpatient	Household size, age, education, health insurance
44.	Sheikh et al., 2022, Bangladesh	To estimate disease-specific incidence of distress financing and catastrophic OOP for hospitalization	Secondary data, data (HIES 2016–2017), Cross-sectional	Two-stage stratified cluster sampling	45,423 Household	Disease-specific (Multiple)	Inpatient	Gender, hospitalization, NCD, cancer, type of provider, rural-urban residence Gender disparity is considerable.
45.	Si et al., 2019, China	To measure the incidence, intensity, and inequality of CHE	Secondary data, National Health Service Survey, 2008, and 2013 Cross-sectional,	Multistage stratified random sampling	1,749 households	Hypertension	Both inpatient and outpatient	*Economic status, number of elderly adults (aged 65+)*
46.	Thuong et al., 2020, Vietnam	To examine the determinants of CHE, specially health insurance	Secondary data (Vietnam Household Living Standard Survey, 2016), Cross-sectional	Stratified Random Sampling	7173 Household	General health care	Both inpatient and outpatient	Age and employment status of household head, severity of illness and injury, type of care utilized, household size
47.	Wang et al., 2023, China	To assess the link between the multi-morbidity of NCD and CHE	China Health and Retirement Longitudinal Study (CHARLS), 2011–2018	Multistage Stratified Probability sampling	17,708 Participants aged 45+	NCD	Both inpatient and outpatient	Multi-morbidity (+)
48.	Wang et al., 2015, China	To measure the extent, determinants, and inequality of CHE	Secondary data, China Health and Retirement Longitudinal Study (CHARLS), 2011	Multistage Stratified Probability sampling	Aged 45+	Chronic disease	Both inpatient and outpatient	*Poverty, Members having multi morbidity, health insurance, type of care utilized, health insurance* *n*
49.	Yadav et al., 2021, India	To measure the impact of out-of-pocket payment and the financial burden due to morbidity events	Quantitative, Secondary data 75th round of National sample survey, cross-sectional	Multistage random sampling	92,527 Household	Multiple	Both inpatient and outpatient	*High OOP, type of provider), type of disease (cancers, cardiovascular diseases, psychiatric conditions, injuries, musculoskeletal, and genitourinary conditions)*,
50.	Zhao et al., 2022, China	To examine the socioeconomic and rural-urban differentials in treatment, health service utilization and CHE	China Health and Retirement Longitudinal Study (CHARLS), 2011, 2015	Multistage Stratified Probability sampling	602 individuals	Cancer	Both inpatient and outpatient	*Outpatient visits, hospitalization, chemotherapy and surgery*
51.	Zhen et al., 2018, China	Compare CHE among two provinces and their determinants	Primary Data, cross-sectional	Multistage stratified cluster random sampling	1,598 Households	General health care	Both inpatient and outpatient	Employment status of the household head, economic status, members with chronic illness, number of inpatient members in the family

Before synthesizing the predictors of CHE with the socio-ecological model, we present some numerics from the World Bank related to financial protection against catastrophic health expenditure in Table 4. We have presented the data of the countries in which we have included literature in this review.

**Table 4:** Numerics related to catastrophic health expenditure

Country	Population aged 65+ (%)	Current health expenditure as % of GDP	OOP as % of Current health expenditure	Domestic General Government Health expenditure as % of GDP	Social health insurance as % of current health expenditure	PHC (Government and donors) as % of GDP
Bangladesh	6	3	74	0	0	0
Brazil	10	10	22	5	1	Not available
China	14	6	35	3	35	1
India	7	3	51	1	7	1(2018)
Iran	Not available	5	37	3	30	Not available
Kenya	3	4	24	2	14	2
Malawi	3	5	20	2	00	3
Malaysia	8	4	36	2	1	1
Mongolia	5	5	27	3	26	Not available
Nepal	6	5	54	2	2	Not available
Nigeria	3	3	75	1	1	Not available
Pakistan	4	3	55	1	1	Not available
South Africa	6	9	5	5	0	Not available
Vietnam	4	5	40	2	32 (2018)	Not available

Source: The World Bank, 2023

If we concentrate on the numerics, the World Bank data on the countries included in the literature shows that Nigeria has the highest OOP. Patients have to pay 75% of their current health expenditures out of their pocket. Bangladesh has the second highest OOP (74%). Conversely, South Africa has the lowest OOP (5%) and Malawi has the second lowest (20%). Brazil spends 10% of its GDP on health, whereas India and Bangladesh spend only 3% on health. To achieve universal health coverage, nations must invest at least four to five% of their GDP in health [16]. The data depicts that countries investing more in health have lower OOP. Social health insurance coverage in current health expenditure is 35, 34, and 30% for China, Vietnam, and Iran, respectively. However, in Bangladesh, the social health insurance coverage is 0%. According to the World Bank data, countries with higher social health insurance coverage have lower OOP.

Primary health care expenditure by government and donors as a percentage of current health expenditure is 3% for South Africa and Malawi but 0% for Bangladesh and Nigeria [17]. Nevertheless, to combat NCDs, resource-poor countries have no other way but to follow the proverb ‘prevention is better than cure’ as the health system is lagging in technology, health professionals, and financial ability to provide curative care for NCDs like cancer [18]. Preventive care is a part of primary health care. But here data from Bangladesh and Nigeria revealed that the government or donor support on primary health care is zero. In China, 14% of the population are aged 65+. In Brazil, it is 10%, for Malaysia and Bangladesh, the percentage is eight, and six respectively. We have to concentrate to face the challenges of aging, have to prepare to meet the health needs of the senior citizens. These are only a glimpse of the neglected health sector investment of the countries that have been reviewed.

### Socio-ecological synthesis

We classified the determinants of CHE and analyzed them in a precise way. We classified the determinants as the demographic, cost-related, and utilized care-related variables, variables that are the constructs of the health belief model, like perceived severity of illness. There were also some healthcare regulation-related variables. Now, we will make a socio-ecological synthesis of the predictors of CHE. If we want a sustainable solution, any problem must be studied from upstream, not downstream. In that case, the socio-ecological model could facilitate the identification of the predictors of CHE from the upstream, as this model encompasses a holistic view of any health problem.

#### Interpersonal predictors

Among the personal attributes, age, sex, education, income, and place of residence are consistent demographic predictors of CHE. With age, susceptibility to disease increases [10, 19]. Old age dependency makes the situation difficult for senior citizens, especially where the social safety nets are unavailable or poor [20, 21]. The patient’s gender is another deciding factor in accessing health care and significantly impacts CHE [3, 22–26]. A study from Bangladesh denotes that the health needs of women are often neglected by their families [25, 26]. In the case of pregnancy-related care, a study from Bangladesh found that facing any health complexity during delivery considerably incurs CHE [27]. Education is associated with CHE as education impacts health-seeking behavior like food habits, health precautions, preventive measures, and choice of care [4, 18, 19, 23, 28].

As a predictor of CHE, income has been studied for different quintiles, and the extent of the relationship varied for different quintiles. A study from Malawi and Nigeria concluded that the better off face higher CHE than people with low incomes [5, 29]. On the other hand, studies from India and Bangladesh concluded that the poor face higher CHE than the rich [21, 30, 31]. Economic status highly dictates the care utilization pattern, but this variable also gave an assorted result. Studies from China, Vietnam, and Nigeria revealed that people with high economic status faced higher CHE than those with lower economic status [29, 32–34]. In contrast, a study from China concluded that people with lower economic status faced higher CHE [35]. The susceptibility to chronic diseases increases with age, which requires regular and long-term care [35]. Nevertheless, the elderly who do not have any earning source at this age, and this dependency pushes them into CHE [36]. Again, several studies from Bangladesh and South Korea found that the loss of income due to disease, an indirect cost of illness, increases CHE incidence [13, 15, 37, 38]. With the increase in sick days, this indirect loss increases, which worsens the financial catastrophe [30].

Now, let us move on to disease-specific factors. Type of illness is a significant predictor of CHE [7, 22, 39]. Studies from Bangladesh, South Korea, India, and China found that diseases that require inpatient care have higher CHE incidence as with the increase in hospitalization, the direct cost and indirect income loss due to disease increases the financial burden [13, 15, 18, 26, 30, 37, 38, 40]. Studies from Bangladesh, China, Vietnam, and Malaysia concluded that NCD, Cancer, and Chronic disease have higher CHE incidence as these diseases demand long-term care and the treatment is expensive [4, 18, 24–26, 32, 41]. Perceived severity of illness and patients/diagnostic delay are two health belief model constructs. Studies from China and Vietnam identified these two as significant predictors of CHE [36, 42]. A study from China on tuberculosis depicted that patient/diagnostic delay, which is the gap between the onset of the symptoms of the disease to the clinical diagnosis, considerably deteriorates treatment outcomes, demands inpatient care, and results in CHE [43].

Rural-urban differences in availability and quality of health care are significant, so the place of residence has been identified as the most consistent predictor of CHE in studies from China, Bangladesh, India, Nepal, Malaysia, Malawi, and Nigeria [4, 5, 13, 14, 21, 22, 26, 27, 31, 41, 42, 44–47]. Disease pattern highly influences the health care utilization pattern and has CHE impact. For instance, type of illness. Studies from China, Bangladesh, Malaysia, and Kenya showed that patients having chronic disease, NCD and cancer face higher CHE [25, 26, 31, 32, 41, 48, 49].

#### Relational predictors

Household composition highly influences the health-seeking behavior of a patient. First comes the type of family, nuclear or extended. Two studies from Bangladesh and South Africa concluded that nuclear families face higher CHE incidence than extended families [3, 44]. The household size gives mixed findings. Studies from China and Vietnam showed that CHE is lower among families with large households [4, 19, 35, 36]. However, a study from Bangladesh found that larger households face higher CHE than smaller ones [31, 37]. The characteristics of the household head are consistent determinants of CHE. Studies from Nigeria, Kenya, India, and Vietnam concluded that the family faces lower CHE if the household head is male, employed, and educated [20, 22, 36, 50]. A comperative study among two provinces of China concluded that if the household head is unemployed, the family faces a highe CHE [51]. On the contrary, families where a female is the breadwinner face higher CHE [4, 20, 22, 50]. Studies from China, Pakistan, Iran, and Vietnam revealed that families with elderly (aged 65+) have higher CHE incidence [4, 32, 33, 47, 52]. However, a study from Nigeria identified having the elderly as a weak predictor of CHE [22]. Studies from China, Nepal, Korea, and Kenya concluded that families having members with chronic disease face higher CHE [20, 34, 42, 53, 54]. Again, the study from Kenya, Pakistan, and South Africa found that families with children have higher CHE incidence [3, 20, 52]. However, a study from Nigeria concluded that the number of elderly or children is a weak predictor of CHE [22]. Social networks have been identified as a defending factor against CHE in studies from Bangladesh, Vietnam, and South Africa [3, 36, 37]. Membership in any social safety net could save people from CHE in Kenya [20].

#### Institutional predictors

Institutional factors attract the highest concentration in the existing literature, where the cost and utilization pattern got priority. OOP is the dominant CHE predictor and is consistent in the existing literature. Studies from China, Mongolia, and Bangladesh concluded that high OOP is the leading cause of CHE [7, 13, 14, 55]. OOP is the payment that the care receivers must pay at the point of care, which no third party reimburses. Two studies from Bangladesh found OOP regressive [13, 14]. Different studies chalked out different causes of high OOP. Physician fees are a significant predictor of CHE in Bangladesh. In contrast, outpatient visits have been identified as an important predictor of CHE in China [14, 40]. The cost of medicine is the main contributor to OOP in Bangladesh, China, and Vietnam [14, 25, 32, 37, 43, 56]. Diagnostic costs considerably increase OOP in Bangladesh and China [37, 43, 56].

Apart from those direct costs, a study in Bangladesh identified indirect costs like food, lodging, and transport costs as significant predictors of CHE [57]. Health-related income loss is another indirect cost that notably impacts impoverishment in South Korea and Bangladesh [13, 15, 37, 38]. The included literature identified predictors related to utilized care. The type of provider plays a dominating role in CHE. Public providers offer treatment at a considerably lower cost than private providers. Studies from Nigeria, Kenya, Bangladesh, and India found that patients accessing care from public providers have a lower risk of CHE than those accessing care from private providers [7, 20, 22, 26, 29, 31, 50, 57]. Again, accessed care is another influential predictor of CHE. Studies from Iran, China, and Vietnam concluded that those who are taking inpatient care very likely face CHE [4, 36, 47, 48, 54]. Moreover, diseases for which the treatment cost is higher have higher CHE. Two studies from China and one from Malaysia found that the cost of chemotherapy and surgery incurs a two-fold increased risk of CHE [40, 47, 58]. A study on tuberculosis in China found that the cost of ancillary services for liver protection increases the threat of CHE [43].

The review identified three spots at the institutional level. Studies from Bangladesh, India, Nigeria, and Kenya found a significant gap in treatment costs between public and private providers [7, 20, 29, 31, 50]. Again, a study from Bangladesh revealed that the public sector needs to be more functional, especially in the underserved areas, which significantly impact CHE [39]. Again, the same study found that private facilities were unavailable in rural coastal areas, which made health care inaccessible to many. Health insurance has been identified as a protector against CHE in several studies from China, Bangladesh, Malaysia, Kenya, and Vietnam [4, 20, 24, 48]. On the contrary, studies from China found that delayed or ignored inpatient treatment coverage often fails to protect patients from CHE [43]. Another study from Pakistan found that the sub-performance of drug regulatory authority is one of the leading causes of the high price of medicine, which is a risk factor for CHE [14, 59].

#### Community predictors

The review found three community-level factors: comorbidity, poverty, and social stigma. A study from Bangladesh argued that the poor cannot pay for the health shocks. Moreover, the income loss due to illness makes them more vulnerable as they have no other source but to go for distress financing, which increases the intensity of poverty [13, 14]. Studies from Bangladesh and Vietnam found that due to poverty, the incidence of forgone care is significant, especially for NCDs and cancer [14, 25, 31, 32]. Social stigma about disease increases the risk of facing CHE, which has been concluded in studies from Bangladesh and Vietnam [36, 37]. The relative prevalence of natural disasters has been found to significantly impact the incidence of CHE in Bangladesh [39].

#### Policy level predictors

**Fig. 3:**
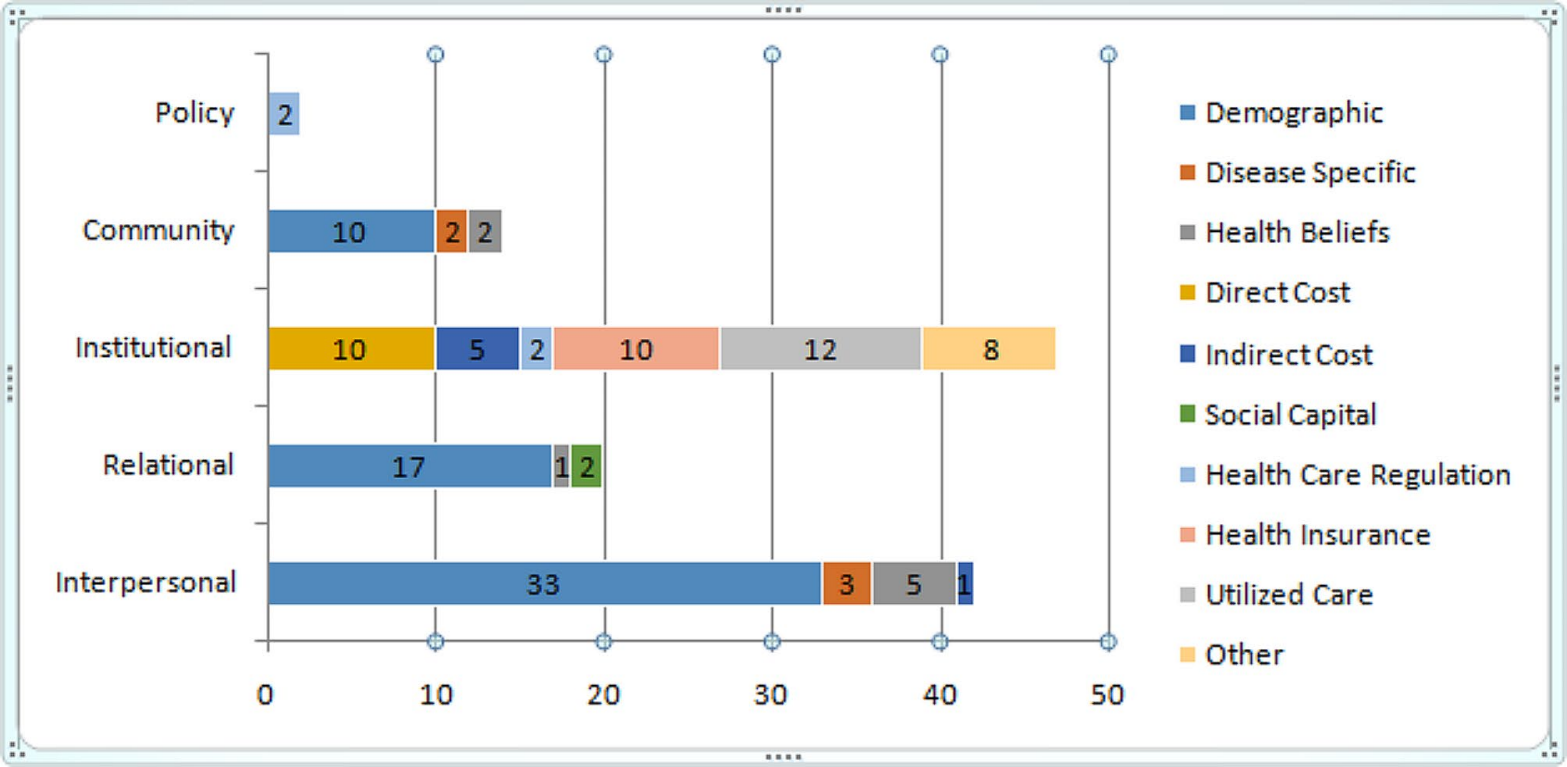
Socio-ecological synthesis of the predictors of CHE

At the policy level, this review has found only a single predictor. Studies from Bangladesh concluded that due to the lack of regulation of health care market, OOP is considerably high, resulting in CHE [10, 39]. The predictors of different levels are summed up in Fig. 3. The details of the predictors and their occurrence in the included literature are summed up in Table 5, where the number in the parentheses refers to the number of the literature as of cited in the reference. Figure 3 presents the gap in the reviewed literature to understand the socio-ecological synthesis better.

**Table 5:** Socio-ecological synthesis of the predictors of CHE

Interpersonal	Relational	Institutional	Community	Policy
Age (20,29,31,41,46,47)	Type of family (2,39)	Out-of-pocket payment for health (17,44,45,59)	Poverty (6,9,12,23,26,35,37,42,44,52)	Unregulated market (8,46)
Gender (4,20,38,39,41,48)	Household size (15,31,43,47,50)	Physician’s fee (45)	Comorbidity (15,51)	
Ethnicity (29)	Gender of household head (4,10,14,31)	Outpatient visit (61)	Social stigma (14,59)	
Education (9,20,31,42,47)	Age of household head (10,50)	Cost of diagnosis (12,18,22)		
Occupation (20)	Education of household head (4,7,10,14,21)	Cost of medicine (27,12,18,22,41,45)		
Income (11,15,20,19,21,28,29,31,32,36,37,60)	Occupation of household head (50,62)	Direct Cost: Cost of ancillary health services (18)		
Economic status (3,15,27,49,62)	Income of household head (7,21)	Food, lodging and transport cost (24)		
Demographic: Marital status (12)	Self-assessment of household head regarding the severity of illness (19)	Income loss of the patient due to disease (1,12,25,44)		
Place of residence (Rural-urban differential) (2,4,19,21,23,26,29,31,32,37,43,45,44,48, 60)	Number of elderly (65+) (2,4,7,27,31,49,60)	Type of provider (Public-private) (3,4,10,14,23,43,48,59)		
Old age dependency (37)	Number of children (7,10,39)	Type of utilized care (inpatient-outpatient) (31,35,50,52,60)		
Type of illness (4,8,59)	Number of people with chronic illness (10,11,21,52,62)	Surgery (6,61)		
Sickness days(36)	Social network (39,50)	Chemotherapy (61)		
Perceived severity of illness (21,50)		Hospitalization (36,42,48,61)		
Patient’s/diagnostic delay(12,18)		Health insurance (10,18,20,31,35,38,47,50,52,62)		
		Delayed or ignored inpatient treatment coverage from insurance (18)		
		Nonfunctioning public facilities (8)		
		Unavailability of private facilities (8)		
		Regulation: Sub-performance of drug regulatory authority (16)		

**Fig. 4:**
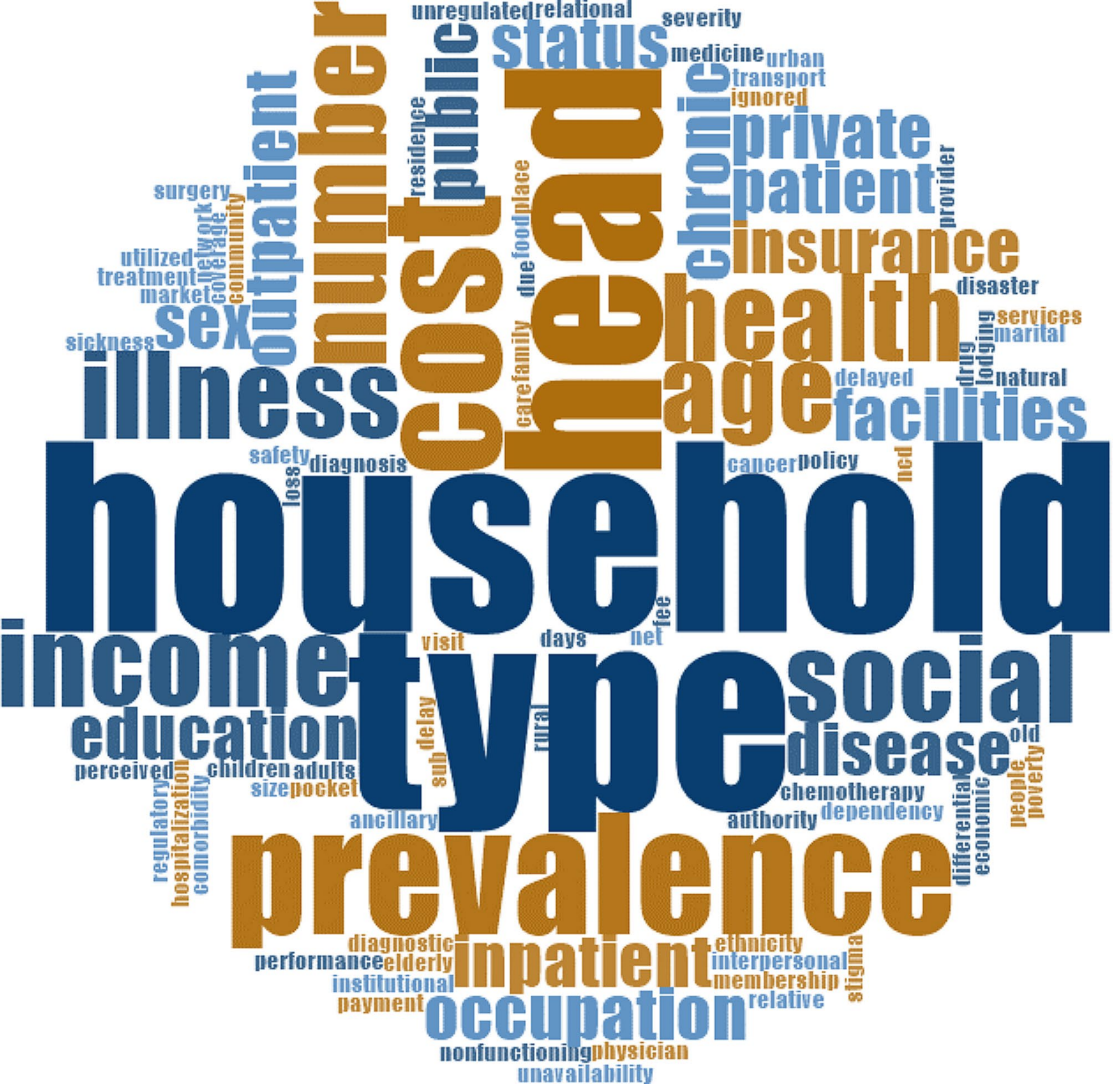
Word cloud depicting word’s relative relevance in the reviewed papers

We presented a word cloud based on all the articles included in the study using NVivo 12. Figure 4 presents the word cloud. The word cloud is a frequently used tool in qualitative studies to visualize the respondents’ responses and facilitate thematic analysis. This is not so suitable to quantitative studies.

In this review, our motivation of using word cloud is different. We first used the word cloud to figure out the relevance of the searched literature with the study objective and to show the relative relevance of the words that occurred in the included literature. Bigger and bolder words have a higher frequency in the reviewed documents. ‘Household’, ‘type’, ‘head’, ‘prevalence’, ‘illness’, ‘health’, ‘income’, and ‘social’ largely occurred in the included literature. The main query of the included papers was to identify the predictors of CHE. The word cloud depicts that the search was quite relevant to the study objective, as all the words that have been visualized in the cloud have close relevance with the predictors of CHE. The bigger and bolder words are mainly relational, interpersonal, and institutional factors, the policy-level factors were smaller.

## Discussion

The objective of this review is to make a socio-ecological synthesis of the predictors of CHE. The included literature mainly dealt with the interpersonal, relational, and institutional predictors and the papers put nominal concentration on community and policy-level factors. The institutional level yielded the greatest number of predictors; however, all of them were solely based on patient responses from the demand side. The supply side predictors, i.e., the providers’ response, were completely missing in the included literature. However, the studies presented altered dimensions of CHE. A study from South Korea revealed many blind spots in the country’s healthcare system, such as chronic kidney disease [53]. The prevalence of such diseases is considerably increasing. Nonetheless, the treatment is minimal against demand and highly expensive, having a high CHE impact. However, most countries’ health systems failed to identify the financial catastrophe and the urgency of treating such fatal diseases at a lower cost.

A study from Vietnam concluded that households with higher socioeconomic status utilized care for NCDs, but patients from lower economic status often forgo care as treatment is expensive [32]. Studies from China and Malaysia showed that in the case of surgery and chemotherapy, patients have a two-fold high risk of facing CHE [40, 58]. Complications during birth have a high incidence of CHE in Bangladesh. Families rarely go for distress financing (donation, sale of assets, loan). Instead, they try to mitigate from their income or savings. The authors implicate these findings in an assorted way [27]. The study argued that this finding might have two reasons. Family ties work positively here, and the family saves money for the upcoming birth event. Conversely, there are also incidents where even if birth becomes complicated, the family does not go to the clinics, and the result might be the loss of life of the mother or the newborn.

A study from Bangladesh found that if the patient is male and earning, distress financing is common for NCDs. However, suppose the patient is female or elderly or unemployed. In that case, the family rarely goes for distress financing instead of forgoing care [25]. A study from China concluded that the assessment of a household head regarding family member’s health status plays a pivotal role in the healthcare utilization pattern of family members [45]. A study from Bangladesh showed that women often could not access formal care due to the lack of financial support, absence of peers to accompany them, or lack of permission from the family, even in cases of NCDs like cancer [25]. Studies on tuberculosis in Bangladesh and China concluded that the delay from symptom to diagnosis (patients/diagnostic delay) deteriorates the treatment outcome [37, 43].

Social stigma is a predictor of CHE. A study from Bangladesh found that in fear of losing a job or being marginalized, tuberculosis patients do not go for clinical diagnosis. Studies from China, India, and Bangladesh concluded that delayed diagnosis increases the risk of hospitalization, a consistent contributor to OOP and the resultant CHE [18, 26, 30, 40]. The literature gave mixed results on economic status and poverty in CHE. Studies from Malawi and Nigeria showed that CHE incidence is higher among the better off as they are more cautious, and their healthcare utilization rate is higher than that of those with low economic status [5, 29]. A study from Bangladesh also concluded that people from lower income quintiles have lower CHE as the utilization of formal care is low [31]. Conversely, poverty consistently predicts CHE, indicating that poor people have higher CHE [13, 18, 21, 27, 28, 37, 46, 48, 54, 58]. To safeguard poor people from financial catastrophe, several studies from China, Kenya, and Malaysia emphasized on health insurance [19, 20, 36, 54]. However, countries where health insurance is available reported that due to insufficient coverage and the delay in reimbursement, health insurance could not save people from CHE [43]. Again, in Mongolia, despite having high social health insurance coverage, the incidence of CHE is high as social health insurance can not cover the treatment cost of the elderly, especially those with chronic diseases [55]. The study from China concluded that basic health insurance has an insignificant impact on CHE, especially for poor people with chronic disease [34]. Another study from China concluded that social health insurance coverage could not save the elderly from facing catastrophic health expenditures [62].

Studies from China and Vietnam identified family ties as a protector against CHE [35, 36]. The papers argued that in large families, family members care for each other, give mental and spiritual support, and have a more extensive social support network and better risk management capacities against CHE.

OOP is the crucial determinant of CHE, and utilized care is the key contributor. Only three studies from Bangladesh and South Korea concentrated on inpatient care, and a study from Pakistan worked on medication [15, 26, 38, 59]. One paper from Bangladesh on spinal cord injury worked on community-based home care [57]. The rest of the paper does not consider the utilized care. Instead, they consider general health care. This is a considerable gap. The existing literature lacks an analysis of the institutional factors contributing to increased diagnosis and medicine costs, as well as the vulnerabilities faced by the poor due to the utilization of care. The reasons behind these issues, particularly in the context of Bangladesh, have received minimal attention in research.

Most papers used secondary data, whereas disease-specific studies used primary data. Studies using secondary data on general health care poorly apprehended the vulnerabilities of a disease. Instead, concentrating on disease and using primary data could help to capture disease-specific vulnerabilities more intensively. Suppose the countries want to identify the causes of high OOP. In that case, they must concentrate more on the healthcare utilization pattern with age, income, and disease prevalence. If we focus on similar studies from the developed world, like Canada, the USA, the UK, or Australia, the authors are concentrating more on supply-side predictors. They emphasize health rights and health-related laws and concentrate more on the community, institutional, and policy levels. However, these aspects are almost absent in the included literature. Only two studies from Bangladesh identified a lack of regulation in the healthcare market, nonfunctional public facilities, and the unavailability of private facilities as predictors of CHE [14, 39].

The WHO proposed health insurance as a safeguard against CHE, which is largely overlooked in the studies from Bangladesh as her insurance coverage is only 2% [63]. On the contrary, studies from China, South Korea, Vietnam, and African countries like Nigeria or Kenya concentrated more on finding a suitable health insurance package based on age or disease prevalence. Social stigma or the need for social networks is studied in countries like South Korea, China, Vietnam, Kenya, and Nigeria. However, Bangladesh is lagging behind in addressing community predictors.

The review found sufficient predictors from the demand side in explaining why does CHE vary across the regions. However, the supply side predictors, more specifically the community and policy level factors, are rare in the included literature. The world bank data revealed that, in the countries of the reviewed literature, OOP varies on an extensive range; domestic general government health expenditure or government and donors support for primary health care vary. Some countries have no or minimal social health insurance coverage. Thus, it is worth investigating the reasons behind such variation. Results from our review suggest that the determinants behind the variations are originated from the loopholes in the supply side determinants at policy level, which are understudied. Furthermore, the difference in government health expenditure is the primary cause of the variation in financial protection against CHE [31]. We have to find out the reasons behind this deficiency. The reasons for high OOP or CHE are partially examined. We have to conentrate on the supply side predictors of CHE. If we concentrate on China, India, South Korea, the studies from those countries used different registers to study the health needs at different ages or diseases. However, many other countries like Bangladesh do not have any age- or disease-specific data sources that the researchers could use. That might be the primary cause of high CHE incidence in the LMICs [9].

## Conclusion

The health-seeking behavior of a person is influenced by his attributes, the family and peers with whom he lives, the institutions with whom he/she interacts, the community where he/she belongs, that shapes his/her culture and values, and the policies that control and, at the same time, facilitate his/her deeds. The health system must consistently address the factors encompassing the five levels and keep coherence among different levels. Any gap among the different levels will worsen the CHE scenario. Nevertheless, this study found significant gap in the studies of the predictors of CHE from two specific aspects. First, the studies mainly concentrated on interpersonal, relational and institutional factors. The community and policy-level factors are rarely studied. The second gap is that the predictors have been studied from the demand side. A study on supply-side predictors is missing in the review; all the studies were on patients, and there is not a single study from the provider side. Saving people with low incomes from financial catastrophes is one of the targets of SDG 3. To achieve this target, countries must fill the gap.

### Implication for research

The review found that authors from resource-poor countries concentrate less on community and policy-level factors. Along with the community and policy level factors, the institutional factors must be examined from the supply side. Health professionals like doctors, nurses, and hospital administrators would better explain the cause of high consultation fees, high price of medicine and diagnosis, high hospital charges, high cost of chemotherapy or surgery, or the reasons for the unavailability of treatment in underserved areas, or, why the access of a patient is denied due to the lack of financial ability, definitely in a more comprehensive way than a patient. The demand for health care is supplier-induced. The patient does not know what they need, and they do not know the utility that they will gain from treatment. So, we have to get the supply-side response.

### Policy implications

To safeguard people experiencing poverty from CHE, the WHO promoted health insurance to protect against financial catastrophe. However, study findings from different countries concluded that insurance coverage is insufficient for many countries, especially to cover the treatment cost of chronic diseases like chronic kidney disease, especially for the elderly. Again, many studies found that large family sizes act as the first line of defense against CHE. Strengthening the family bondage could save people from CHE. The social stigma of the disease has a more significant mental effect with visible physical complexity. The studies do not care much about the social stigma of a disease. However, the cost of CHE is not only a financial issue but also has immense social implications. Countries must increase coverage and design insurance benefit packages that consider age, income, disease, and treatment type. Again, the impact of social capital must be recognized by the policymakers as a shield against CHE and how this could be better utilized against CHE.

### Limitations of the study

To the best of our knowledge, this paper is the first attempt to synthesize the predictors of CHE with the socio-ecolofgical model. The findings may be helpful to chalk out the loopholes of the health system more precisely that pushes people to catastrophic health expenditure.

The study has some limitations. Most of the included documents used secondary data sources and worked on general health care to measure the incidence of CHE. We have to concentrate more on primary data-based and disease-specific studies to capture the accurate predictors. In the online data sources, such literature is limited on LMICs. That is why this systematic review’s proportion of secondary data-based studies was higher. This is a limitation of the study. Again, we excluded non-English documents from the review, and the data search did not cover the data from some other related electronic data sources like Pub-med. We did not go for any open search like google scholar. Our study was also confined to LMICs. The inclusion of data from more data bases and open sources, and the non-English sources may produce more comprehensive results and may be a scope for future research.

## Data Availability

The dataset analyzed during the current study available from the corresponding author on reasonable request.

## Abbreviations

CHE: Catastrophic Health Expenditure
GDP: Gross Domestic Product
LMIC: Lower Middle-Income Country
NCD: Non-communicable Disease
OOP: Out-of-Pocket
PHC: Primary Health Care
PRISMA: Preferred Reporting Items for Systematic Reviews and Meta-Analysis
SDG: Sustainable Development Goal
WHO: World Health Organization
